# Application of artificial intelligence in hypertension

**DOI:** 10.1186/s40885-024-00266-9

**Published:** 2024-05-01

**Authors:** Jung Sun Cho, Jae-Hyeong Park

**Affiliations:** 1 Division of Cardiology, Daejeon St. Mary’s Hospital, College of Medicine, The Catholic University of Korea, Seoul, Republic of Korea; 2 Catholic Research Institute for Intractable Cardiovascular Disease, College of Medicine, The Catholic University of Korea, Seoul, Republic of Korea; 3 Department of Cardiology in Internal Medicine, Chungnam National University, Chungnam National University Hospital, 282 Munhwa-ro, Jung-gu, 35015 Daejeon, Republic of Korea

**Keywords:** Artificial intelligence, Machine learning, Hypertension, Disease management

## Abstract

Hypertension is an important modifiable risk factor for morbidity and mortality associated with cardiovascular disease. The incidence of hypertension is increasing not only in Korea but also in many Western countries due to the aging of the population and the increase in unhealthy lifestyles. However, hypertension control rates remain low due to poor adherence to antihypertensive medications, low awareness of hypertension, and numerous factors that contribute to hypertension, including diet, environment, lifestyle, obesity, and genetics. Because artificial intelligence (AI) involves data-driven algorithms, AI is an asset to understanding chronic diseases that are influenced by multiple factors, such as hypertension. Although several hypertension studies using AI have been published recently, most are exploratory descriptive studies that are often difficult for clinicians to understand and have little clinical relevance. This review aims to provide a clinician-centered perspective on AI by showing recent studies on the relevance of AI for patients with hypertension. The review is organized into sections on blood pressure measurement and hypertension diagnosis, prognosis, and management.

**Graphical Abstract:**

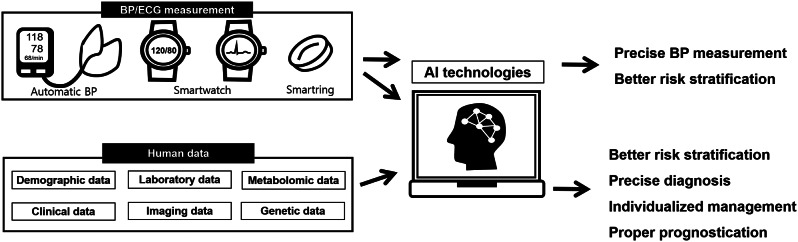

## Introduction

Hypertension is a highly prevalent chronic disease affecting approximately 1.28 billion people worldwide [1]. Despite increases in awareness and treatment rates, hypertension and its complications are still a significant clinical burden [1, 2]. This may be because hypertension is a heterogeneous phenotype caused by many factors, including age, sex, body mass index, lipid profiles, personal habits (stress, smoking, physical activity, etc.), socioeconomic status, environment, and genetics [2–4].

Artificial intelligence (AI) may represent a breakthrough in understanding and treating hypertension and an important tool for overcoming the current problems associated with managing patients with hypertension. AI is increasingly impacting our daily lives, not only in the areas of advertising, finance, law, and education but also in medicine, where AI technologies are now being applied to the field of hypertension. However, AI research is limited to exploratory techniques with minimal clinical implications and can be difficult to understand for clinicians who are not experts in machine learning (ML) or deep learning (DL).

Before generating this review article, we conducted a detailed search of journal databases, including PubMed, EMBASE, and Web of Science, for applications of AI in hypertension from 2015 to 2023. Search terms included AI, artificial neural network, DL, ML, hypertension, blood pressure (BP), and BP measurement. We included clinical trials involving hypertensive patients rather than healthy individuals. To narrow our focus, we excluded exploratory technical studies, validation studies, and community-based primary health care studies. In this article, we review recently published studies of AI applications to BP measurement, hypertension diagnosis and prognostication, and management of hypertensive patients to clarify the current clinical implications of AI in hypertension from a clinician-centered perspective.

### Clinical implications of AI

#### BP measurement

Accurate BP measurement is a cornerstone of hypertension diagnosis and management. The originally recommended method for measuring BP is the oscilloscopic method [2]. Noninvasive BP estimation devices include the traditional sphygmomanometer using an inflatable cuff, stethoscope, and manometer using Korotkoff sound, which was introduced in 1905 [5, 6]. Nevertheless, it is difficult and time consuming to measure BP. Moreover, oscilloscopic measurement has significant interoperator differences. Compared to the conventional oscilloscopic method, automatic monitors have become popular in recent years because they can easily measure BP and can be used to measure BP at home. Home BP monitoring can be the key to better BP control [7]. To improve the accuracy of BP measurement, many new algorithms or devices have been developed using AI technology to improve the precision, accuracy, and reproducibility of BP measurement. Automatic noninvasive BP estimation devices can be divided into oscillometric, auscultatory, and cuffless methods.

AI algorithms have been used for many years to improve the accuracy of BP measurement with automatic oscillometric BP monitors, and several experimental studies on the performance of these algorithms have been published recently [8]. In several clinical studies, auscultatory waveform studies have been published mainly for inflated cuff-based BP measurement [9]. Furthermore, Chu et al. [10] developed a smartphone app with an auscultatory waveform analysis algorithm to simultaneously evaluate the accuracy of an automated oscillometric BP device.

Recently, noninvasive automated cuff-less BP monitors have been developed and are commercially available [11]. Various methods have been proposed for cuff-less BP estimation, including methods based on the pulse transit time (PTT) and the pulse arrival time (PAT) using photoplethysmograms (PPGs) and electrocardiograms (ECGs) [12, 13]. A PPG is a noninvasive optical recording used to detect changes in blood volume in tissue, particularly in the microvascular bed. The PPG sensor detects changes in the amount of transmitted or reflected light and generates a PPG waveform. The volume and dilation of the arteries can be related to the pressure in the arteries, so the PPG signal produces a pulse waveform similar to the pressure waveform produced by a tonometer. The collected signal and data can be fed into an ML model to obtain estimates of SBP and DBP from the raw signal [13].

The PAT is more commonly used to describe the time from the R-wave of the ECG to the arrival of the arterial pulse at the peripheral site. The PTT, on the other hand, is a more general concept that includes the time it takes for the pulse to travel between two selected points. It is well known that there is a positive correlation between the PTT or PAT and BP because arterial stiffening resulting from increased BP leads to a rise in the pulse wave velocity and a decrease in the PTT [12–14]. These methods are used in cuff-less noninvasive automated BP monitors, which are suitable for taking measurements at any time and can be used for continuous monitoring.

Recently, several clinical studies of multiple DL algorithms for BP estimation using PPGs [15], calibration-free measurement [16], smartphone apps [17, 18], and wrist-worn cuff-less devices [19, 20] have been published. In one study, retinal fundus photographs were used to measure BP, rather than the presence of complications of hypertension, but the accuracy was not as good as a direct BP measurement [21]. Nevertheless, the fact that BP can be measured with imaging data is an example of how various methods of BP measurement may be tested in the future (Table 1).

**Table 1:** Summary of recently published artificial intelligence (AI)-based clinical studies on BP measurement methods conducted in hypertensive patients

	Authors	Years	Results	No. of subjects
**Inflatable cuff based auscultatory measurement**				
AI algorithm could automatically estimate BP using auscultatory waveform	Argha et al. [9]	2020	SBP, MAE = 1.7 ± 3.7 mmHg/ DBP, MAE = 3.4 ± 5.0 mmHg	350
BP monitors by the auscultatory method: A smartphone-based APP	Chu et al. [10]	2017	SBP, MAE = 2.45 ± 1.47 mmHg/ DBP, MAE = 0.69 ± 1.36 mmHg	85
**Cuff less non-invasive measurement**				
A multistage deep neural network to estimate SBP and DBP using the PPG.	Esmaelpoor et al. [15]	2020	SBP, Mean SD = + 1.91 ± 5.55 mmHg/ DBP, Mean SD = + 0.67 ± 2.84 mmHg	200
Calibration free BP estimation with PPG	Samimi et al. [16]	2023	SBP, *r* = 0.73/ DBP, *r* = 0.77	250
Smartphones APP using only HR and modified normalized pulse volume (mNPV) could assess BP	Matsumura et al. [17]	2018	SBP, *r* = 0.685/ DBP, *r* = 0.685	49
A smartphone-case based single-channel ECG monitor simultaneously with a PPG pulse wave recording	Sagirova et al. [18]	2021	The Bland–Altman analysis; SBP, SD = 3.63, and bias was 0.32/ DBP, SD = 2.95 and bias was 0.61/ SBP, *r* = 0.89 DBP, *r* = 0.87	512
Continuous monitoring of BP using a wrist-worn cuff less device	Sayer et al. [19]	2022	SBP, *r* = 0.91, MAE = 8.2 ± 5.8/ DBP, *r* = 0.85, MAE = 6.4 ± 3.9	34
**BP estimation with image modality**				
BP measurement using Retinal fundus photographs	Poplin et al. [21]	2018	SBP, MAE = 11.35/ DBP, MAE = 6.42	Cohort 1 = 48,101/ Cohort 2* =12,026

APP, a smartphone application; BP, blood pressure; DBP, diastolic blood pressure; ECG, electrocardiogram; HR, heart rate; HTN, hypertension; MAE, mean absolute error; PAT, pulse arrival time; PPG, photoplethysmogram; SBP, systolic blood pressure; SD, standard deviation

Cohort 2* was for external validation

### Diagnosing hypertension

Many studies have been published using AI to diagnose [22, 23] or predict the occurrence of hypertension [24–26] in the general population. As this article focuses on studies in hypertensive patients, recent studies that have attempted to use AI to identify subtypes of hypertension in hypertensive patients were reviewed (Table 2).

**Table 2:** Summary for all the diagnosing hypertension based on artificial intelligence (AI) presented in this paper

Title	Authors	Year		Selected Feature		No of subjects	Best performance Algorithms
Model to predict BP over 4 weeks for an individual with high variability of BP	Koshimizu et al. [28]		2020	BP at home every day for 2 years or more; Medical examination data such as gender, age, and others	423		Multi-input multi-output deep neural networks including LSTM or GRU
High BP variability groups based on K means clustering showed higher HR than quartile grouping.	Tsoi et al. [27]		2020	Visit to visit BP variability; two-third from the SBP variation and one-third from the DBP variation	SPRINT study=8133/ HK cohort=1094		K-means clustering
Prediction of masked hypertension and masked uncontrolled hypertension using ML	Hung et al. [29]	2021		Office SBP, DBP, MAP, and PP, betablocker, HDL-c		Cohort 1=970, Cohort 2* =416	Random forest
Prediction of uncontrolled hypertension within the coming three-month period	Mohammadi et al. [59]		2019	EHR data	17,4169		Logistic regression
ML based on clinical parameters and features derived from the ECG, to detect hypertension	Angelaki et al. [31]		2022	Features derived from the ECG, age, BMI, BMI-adjusted Cornell criteria. R wave amplitude in aVL and BMI-modified Sokolow-Lyon voltage	1091		Random forest
Differential diagnosis of secondary hypertension based on DL	Wu et al. [60]		2023	EHR of each patient includes chief complain and present illness, medical examination results	11,961		Feature encoder with additional attention layer
Prediction of Post-treatment ABPM	Hae et al. [61]		2023	Clinical and lab findings, initial ABPM data, anti-hypertensive medication	1,129		Cat-Boost

ABPM, ambulatory blood pressure monitoring; BMI, body mass index; BP, blood pressure; DBP, diastolic BP; DL, deep learning; ECG, electrocardiogram; EHR, electric health record; GRU, gated recurrent unit; HDL-c, HDL-cholesterol; HER, electric health record; HR, Hazard ratio; LSTM, long short-term memory; MAP, Mean arterial pressure; ML, machine learning; PP, pulse pressure; SBP, systolic BP; SPRINT, the Systolic Blood Pressure Intervention Trial

Cohort 2* was for external validation

BP variability (BPV) is known to be an independent risk factor for cardiovascular disease (CVD), but the diagnostic criteria are vague. Tsoi et al. [27] reanalyzed previous randomized controlled trials, including the SPRINT trial, and established patient clusters based on low, medium, and high BPV levels using traditional quantile clustering and 5 ML algorithms. K-means clustering showed the most stable and reliable results. It showed that approximately one-seventh of the population with a high level of BPV correlated with a higher risk of stroke and heart failure [27]. BPV data are time series and address different time scales; thus, Koshimizu et al. [28] predicted BPV using a multi-input multi-output deep neural network. The dataset in this study included cardiovascular risk factors and home BP readings every day for 2 years or more.

**Fig. 1:**
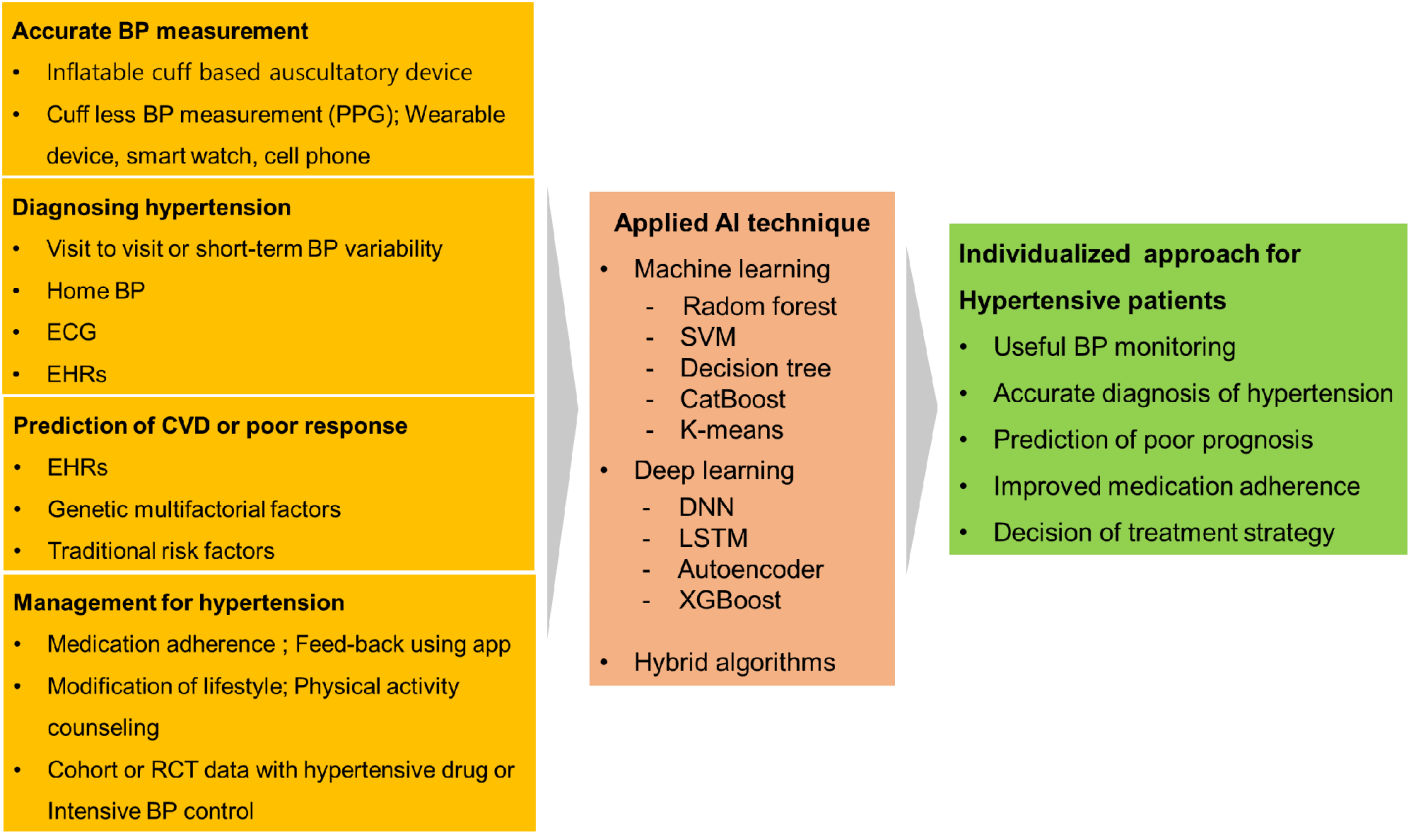
Big data commonly used in each category of hypertension research, and the algorithms that use it to implement individualized approaches for hypertensive patients. BP, blood pressure; CVD, cardiovascular disease; DNN, deep neural network; ECG, electrocardiography; EHRs, electronic health records; LSTM, long short-term memory; PPG, photoplethysmogram; RCT, randomized controlled trial; SVM, support vector machine; XGboost, extreme gradient boost

Recent studies have shown that DL or ML algorithms can be used to diagnose clinically important conditions such as masked uncontrolled hypertension or secondary hypertension using not only big data from electronic health records (EHRs) but also easily identified clinical features such as office BP, pulse pressure, use of beta-blocker, and high-density lipoprotein-cholesterol (HDL-C) level [29, 30]. Furthermore, there have been several attempts to diagnose hypertension using the amplitude and voltage of ECG waves [23, 31] (Fig. 1).

### Prediction of prognosis in hypertensive patients

As BP is a complex multifactorial phenotype that is influenced by many factors, including genomic, demographic, lifestyle and environmental factors, the prognosis of hypertensive patients is also influenced by many factors. In analyzing multimodal data, AI provides the opportunity to conduct integrated analyses of hypertension as a newer analytic method and provides more insight into the prognosis and risk stratification of hypertensive patients [32, 33]. Louca et al. [34] analyzed the multimodal data from TwinsUK with concurrent BP, metabolomics, genomics, biochemical measures, and dietary data. The features tested in this study were then probed using the same algorithm in an independent dataset of 2,807 individuals from the Qatar Biobank. They found that the most predictive features of BP are the traditional risk factors, metabolites, and diet, while genetics (single nucleotide polymorphisms) did not appear to play a major role in predicting prognosis.

Several studies have shown that ML algorithms are better in the prediction of the 10-year risk of cardiovascular events than an established risk prediction approach such as the Framingham score or ACC/AHA CVD Risk Calculator (Table 3) [35–39].

**Table 3:** Summary for all the cardiovascular disease (CVD) risk prediction model in patients with hypertension using AI presented in this paper

Database	Authors	Year	Results	Outcome	Algorithms
Twins genomics, BP, metabolomics, biochemical measures, and dietary data	Louca et al. [34]	2022	SBP, MAE = 11.31 ± 0.64 mmHg R^2^ = 0.39 ± 0.04	Understanding of their in-between relationships and expands the range of potential biomarkers for BP	XGBoost
National Health Information Database^a^	Lee et al. [39]	2022	Accuracy = 0.92, F_1_ Score = 0.92, AUC-ROC = 0.99	Prediction CVD death within a year in patients with hypertension	DNN
508 young patients with hypertension at a tertiary hospital	Wu et al. [30]	2020	AUC-ROC = 0.75	The ML approach was better than that of the recalibrated Framingham Risk Score model	Recursive feature elimination, extreme gradient boosting, and 10-fold cross-validation
35,332 EHRs from hypertensives	Ren et al. [37]	2019	Accuracy = 0.87	Kidney disease prediction in hypertension patients	Hybrid model; BiLSTM and Autoencoder
A 13-year follow up data set from MESA (the Multi-Ethnic Study of Atherosclerosis) of 6459 participants and the FLEMENGHO study (the Flemish Study of Environment, Genes and Health Outcomes) to validate the model in an external cohort	Kakadiaris et al. [36]	2018	Sensitivity = 0.86, Specificity = 0.95, and AUC = 0.92	ML calculator which use the same 9 traditional risk factors Outperforms ACC/AHA CVD Risk Calculator in MESA	SVM
Using data on 423,604 participants without CVD at baseline in UK Biobank	Alaa et al. [38]	2019	AUC-ROC = 0.774	AutoPrognosis model improved risk prediction compared to Framingham score a Cox proportional hazards model	^b^AutoPrognosis,
Prospective cohort study using routine clinical data of 378,256 patients from UK family practices, free from CVD	Weng et al. [35]	2017	Sensitivity = 0.675, PPV = 0.184, Specificity = 0.707, NPV = 0.957	ML improves accuracy of CVD risk prediction over 10 years	

AUC, area under curve; BiLSTM, Bidirectional Long Short-Term Memory; BP, blood pressure; CVD, cardiovascular disease; DNN, deep neural network; EHR, electric health record; NPV, negative predictive value; PPV, positive predictive value; ROC, receiver Operating Characteristic; SVM, Support Vector Machines

^a^age, sex, income, body mass index, BP, variance of smoking, physical activity, lipid profile, and fasting plasma glucose

^b^an algorithmic tool that automatically selects and tunes ensembles of ML modeling pipelines comprising data imputation, feature processing, classification and calibration algorithms

The features most strongly associated with CVD vary across studies, depending on the algorithm or input dataset. Lee et al. [39] reported characteristics such as age, sex, income, body mass index, BP, smoking, physical activity, lipid profile, and fasting plasma glucose level associated with CVD. Lacson et al. [40] reported that age, the urine albumin/creatinine ratio, the estimated glomerular filtration rate, serum creatinine, history of subclinical CVD, total cholesterol, a variable representing time-series SBP signals using wavelet transformation on HDL‐C, the 90th percentile SBP, and triglyceride were significantly associated with CVD. Wu et al. [30] extended predictions from CVD to end‐stage renal disease and all‐cause mortality in 508 young patients by using several clinical variables, namely, the left atrial diameter, HDL‐C level, cholesterol level, big endothelin‐1 level, right arm DBP, right leg SBP, left leg SBP, right leg DBP, left arm SBP, mean nocturnal arterial oxygen saturation, past maximum SBP, and blood urea level.

The prognostication of hypertensive patients is a highly dynamic domain in AI research, leading to the emergence of diverse AI algorithms. Hybrid or multiple algorithms have been applied to improve the accuracy of prognostic models and analyze multimodal datasets, such as those based on EHRs containing textural descriptions and discrete physical indicators [37, 38]. In addition, to overcome biases arising from differences in race/ethnicity or primary/tertiary healthcare systems, existing large international prospective cohorts or randomized controlled trials have been reanalyzed and used for external validation [35, 36].

### Management of hypertension

AI could play a role in novel digital interventions, such as promoting patient awareness, self-monitoring, healthy behaviors, and medication adherence. In other words, AI can be integrated into health coaching apps that automatically analyze patients’ BP or activity data from wearable BP devices and/or social media and then provide personalized feedback, including suggestions for BP medications and lifestyle modifications. Recently, randomized clinical trials (RCTs) of mobile apps for managing BP have been reported. One RCT found a small improvement in self-reported adherence with no change in SBP compared to the control group when using a smartphone app to improve medication adherence [41]. However, another RCT utilizing a mobile self-monitoring BP app in conjunction with a feedback algorithm showed a significant improvement in BP control [42].

AI could be useful for personalized hypertension management in terms of antihypertensive drug selection or BP control strategies, such as intensive BP control. Several RCTs have demonstrated the beneficial effects of intensive BP control, and recent ACC/AHA hypertension guidelines recommend intensive BP control [3, 43, 44]. However, intensive BP control may sometimes be difficult to apply to all patients. Therefore, it is important to define which patients can benefit from intensive BP control. AI can be used to define personalized benefit through phenotypic representation in clinical trials. Oikonomoun et al. developed a practical tool for individualized selection of intensive versus standard SBP control in patients without and with type 2 diabetes mellitus [45]. In addition, a study was recently conducted for personalized optimal antihypertensive drug selection. Data mining methods utilizing data from successful and unsuccessful cases were applied to reveal the spectrum of clinical characteristics or important clinical attributes (sensitizers) of five commonly used drugs (irbesartan, metoprolol, felodipine, amlodipine, and levo-amlodipine) [46].

By analyzing big data using ML, Koren et al. [47] showed that drug options that are not reflected in the latest guidelines, such as beta-blockers, proton pump inhibitors and statins, improve the success rate of hypertension treatment. This shows that AI can be used to consider new indications for already marketed drugs.

It is commonly assumed that the absolute risk reduction (ARR) of a treatment-induced cardiovascular event is proportional to baseline risk, with the greatest benefit in high-risk patients. Using individual participant data from the SPRINT and ACCORD-BP trials, Duan et al. showed that an X-learner correctly observed that individualized treatment effects were often not proportional to baseline risk. This study demonstrated that ML methods can be used to improve the identification and calibration of individualized treatment effect estimates from clinical trial data [48]. Li et al. [49] evaluated nonadherent patients’ characteristics from the New York City Community Health Survey using the ML segmentation approach as exhaustive chi-square automatic interaction detection. The study revealed that the most significant predictors of nonadherence for young adults aged 18 to 44 years were no diabetes and white, Asian or Hispanic race. Moreover, uninsured status, no diabetes mellitus, and moderate or high neighborhood poverty were predictors of nonadherence among adults aged 45 to 65 years. Older adults aged 65 years and older were more likely to be nonadherent if they had a low household income or lived in neighborhoods with moderate to high levels of poverty.

Population segmentation analysis can help interpret complex interactions or correlations between variables and offer targeted and effective population health interventions for each segment. This approach is more effective than regression analysis.

### Limitations of applying AI in hypertension

There are several challenges to the implementation of AI for managing patients with hypertension. Several regulatory healthcare systems, such as the United States Food and Drug Administration (FDA) and Korean Ministry of Food and Drug Safety, have announced approval guidelines [50–53]. However, there is still a lack of expert consensus or guidelines. In addition, there are legal and ethical concerns about processing large volumes of data. AI research requires large-scale data for training, testing, and validating.

AI algorithms are trained on previous datasets, which can reflect selection bias based on factors such as sex, race, and socioeconomic status. These biases can be encoded into the algorithm, leading to discriminatory outcomes. Therefore, researchers should develop techniques to debias datasets and algorithms, such as using diverse training data and implementing fairness-aware algorithms. One issue associated with AI research is the lack of transparency and explainability. AI models are often opaque and difficult to understand, creating a ‘black box’ problem. This makes it challenging to explain how the algorithm arrives at its decisions, raising concerns about accountability and trust [32, 54, 55]. Researchers should investigate methods to increase the interpretability of AI models. Techniques such as importance maps or attention mechanisms can be used to emphasize the data components that have the most significant impact on the algorithm’s output. Because AI systems often require access to large amounts of personal data to function effectively. This raises concerns about data privacy, security and the potential for misuse. Thus, it is essential to ensure robust data privacy regulations and advocate responsibility in the data collection practice.

Also, an AI algorithm and the accuracy of the algorithm depend on the amount of data. Many AI studies have reanalyzed existing cohorts or published clinical trials to confirm superiority over statistical analysis and gain new insights. There are only a few reliable RCTs in hypertension AI research, such as those assessing mobile apps for hypertension management and new BP measuring devices (Tables 1 and 4). Recently, various AI algorithms have been developed by many institutions. The lack of standardization and interoperability, such as overfitting, is inevitable, although most AI studies validate their algorithms in separate datasets [56, 57]. AI can make decisions about the diagnosis, treatment, and prognosis of hypertension, thereby mimicking a clinician but is not considered able to replace the physician. AI is based on automated learning from data, so if the data are biased, the algorithm could generate the wrong data-driven decision [58]. Because AI draws conclusions in the most efficient way for its purpose without regard to medical ethics, clinicians with some insight into medical ethics or clinical settings must become involved in the development and implementation of algorithms [32].

**Table 4:** Summary for all the management of hypertension based on artificial intelligence (AI) presented in this paper

Title	Authors	Year	Conclusion	Design	No. of patients
Self-Monitoring of BP and feed-back using APP	Choi et al. [42]	2022	Improvement of Home BP and drug adherence	RCT	180
Medication reminder alert and adherence report APP	Morawski et al. [41]	2018	Improvement of drug adherence but not significantly lowering BP	RCT	411
Computational trial phenomaps and ML	Oikonomou et al. [45]	2022	Algorithm for individually selecting patients to benefit from intensive BP control	SPRINT and ACCORD-BP trial	9,361
Characterizing the critical features when personalizing antihypertensive drugs using spectrum analysis and machine learning methods	Chunyu et al. [46]	2020	A data-driven reference for the personalization of clinical antihypertensive drugs	Registry data	14,581
ML techniques such as decision trees and neural networks identified determinants that contribute to the success of hypertension drug treatment	Koren et al. [47]	2018	Beta blocker as a potential promising antihypertensive drug. PPIs and statins have been very recently identified as effective in lowering BP	Israel Health service organization data	30,705
The X-learner as a meta-algorithm specifically designed for estimating individual treatment effects	Duan et al. [48]	2019	ML methods may improve discrimination and calibration of individualized treatment effect estimates from clinical trial data	SPRINT and ACCORD-BP trial	14,094
Decoding Nonadherence to Hypertensive Medication in NYC: A Population Segmentation Approach	Li et al. [49]	2019	Identifying segments of adults who do not adhere to hypertensive medications has practical implications as this knowledge can be used to develop targeted interventions important characteristics that can be used to predict nonadherence behaviors	Hypertensive patient from 2016 New York City Community Health Survey	10.2% of adults in NYC

gACCORD-BP Trial, The Action to Control Cardiovascular Risk in Diabetes blood pressure trial; APP, a smartphone application; AUC, area under curve; BP, blood pressure; ML, Machine learning; NYC, New York City; PPI, proton pump inhibitor; RCT, randomized clinical trial; ROC, Receiver Operating Characteristic; SPRINT, the Systolic Blood Pressure Intervention Trial

## Conclusions

Most AI studies on hypertension have remained exploratory technology assessments or have reanalyzed data from retrospective cohorts or RCTs focused on other topics. To date, only a few RCTs have tested AI algorithms in hypertension, such as algorithms for mobile apps or BP measuring devices. Nevertheless, with the innovative development of AI technology, AI has the potential to overcome the stagnation in hypertension and all aspects of hypertension clinical practice, including BP measurement, diagnosis, prognostication, and management. Collaboration with medical professionals, particularly in the field of hypertension research, is crucial during the development and validation process of the AI model to ensure clinical relevance.

## Data Availability

Not applicable.

## Abbreviations

AI: artificial intelligence
ARR: absolute risk reduction
BP: blood pressure
CVD: cardiovascular disease
DBP: diastolic blood pressure
DL: deep learning
EHR: electronic health records
HDL-C: high-density lipoprotein-cholesterol
ML: machine learning
PAT: pulse arrival time
PPG: photoplethysmogram
PTT: pulse transit time
RCT: randomized controlled trial
SBP: systolic blood pressure
